# Three-Dimensional Morphologic Analysis of Foot Deformities Using Weightbearing CT and Semiautomated Segmentation Technique

**DOI:** 10.3390/jcm15145588

**Published:** 2026-07-16

**Authors:** Chan Kang, Sang Bum Kim, Woo Jin Shin, Sung Jun Moon, Byung-Ki Cho, Sung Hoo Kim, Gi Soo Lee, Jeong Kil Lee, Jae Hwang Song

**Affiliations:** 1 Department of Orthopaedic Surgery, College of Medicine, Chungnam National University, Daejeon 35015, Republic of Korea; faschan@hanmail.net (C.K.); sangbumos@me.com (S.B.K.); gs1899@gmail.com (G.S.L.); earthlee82@cnuh.co.kr (J.K.L.); 2 Department of Orthopaedic Surgery, College of Medicine, Konyang University, Daejeon 35365, Republic of Korea; tlsdnwls94@naver.com (W.J.S.); konyang15@naver.com (S.J.M.); 3 Department of Orthopaedic Surgery, College of Medicine, Chungbuk National University, Cheongju 28644, Republic of Korea; titanick25@naver.com (B.-K.C.); hoo414414@hanmail.net (S.H.K.)

**Keywords:** WBCT, automated segmentation technique, 3D, foot deformity

## Abstract

Recent increases in life expectancy have been accompanied by a growing prevalence of foot deformities, leading to increased clinical interest in their diagnosis and treatment. However, conventional radiography and computed tomography (CT) have inherent limitations in accurately evaluating the complex three-dimensional (3D) morphology of foot deformities, including flatfoot deformity and cavovarus deformity. These limitations may hinder precise characterization of deformity patterns and complicate surgical decision making. To address these challenges, substantial efforts have recently been directed toward the development of advanced imaging and analytic techniques. Weightbearing computed tomography (WBCT) combined with a semiautomated segmentation technique has emerged as a promising modality for the 3D assessment of normal foot anatomy and pathologic deformities. Recent studies have demonstrated the clinical utility of these technologies in improving deformity characterization, quantitative morphologic analysis, and preoperative planning. This review summarizes the current literature regarding 3D morphologic analysis of foot deformities using WBCT and semiautomated segmentation software, provides a comprehensive overview of contemporary imaging-based analytic techniques, and discusses their potential clinical applications and future directions in foot and ankle surgery.

## 1. Introduction

As life expectancy increases and the aging population expands, foot deformities are being encountered more frequently in clinical practice, leading to growing interest in their diagnosis and treatment [1]. Accurate characterization of foot and ankle morphology plays a critical role in establishing diagnosis, planning surgical intervention, and evaluating postoperative outcomes in foot and ankle disorders [2,3]. 

Traditionally, evaluation of the foot and ankle has relied on weightbearing two-dimensional (2D) radiographs, which have long been regarded as the standard imaging modality for assessing osseous alignment [4]. Nevertheless, conventional radiographic images exhibit important limitations when applied to complex foot deformities, such as flatfoot and cavovarus deformities. In particular, overlapping anatomical structures in complex foot deformities, such as bone stacking, may obscure accurate visualization of the hindfoot and midfoot, while rotational abnormalities and true three-dimensional (3D) spatial relationships cannot be reliably quantified using planar imaging alone [5]. As a result, these inherent limitations may reduce diagnostic reliability and adversely affect the accuracy of surgical planning and correction.

Weightbearing computed tomography (WBCT) has substantially improved the evaluation of complex foot deformities by enabling imaging under physiologic weightbearing conditions [6]. In contrast to conventional CT, WBCT allows multiplanar assessment of the foot while the patient is standing naturally. As a result, this modality has recently gained considerable attention as an essential diagnostic tool in foot and ankle surgery, largely overcoming many of the shortcomings associated with conventional CT imaging [6]. Nevertheless, manual assessment of foot and ankle alignment parameters may remain challenging in severe deformities, even with WBCT data, largely because most conventional measurements were originally developed for 2D radiographic imaging and require selection of an appropriate measurement plane within a volumetric dataset [7]. To overcome these limitations, dedicated 3D anatomic modeling software platforms have been developed to enable computerized techniques for image processing [5,8,9]. Several techniques can be selectively applied, including foot–ankle offset, bone segmentation, distance mapping, and coverage mapping [9]. Among these, automated bone segmentation has been most widely utilized in both clinical practice and basic research [5,10]. Previous studies have demonstrated that these 3D measurements provide greater reliability than conventional 2D radiographic assessments. For example, Kvarda et al. [11] reported improved reliability for several important ankle parameters, whereas Sangoi et al. [8] showed that semiautomated 3D analysis more effectively characterizes the complex rotational deformities of feet.

Although several review articles have recently addressed the role and clinical utility of WBCT in the diagnosis and treatment of foot and ankle disorders [6,7,9,12,13], there remains a lack of comprehensive and systematic reviews specifically addressing the clinical applications of semiautomated bone segmentation using WBCT datasets across various foot deformities. The purpose of this review is to summarize the current literature on 3D morphologic analysis of foot and ankle deformities using WBCT-based semiautomated segmentation technique, to provide a comprehensive overview of contemporary imaging-based analytic techniques, and to discuss their potential clinical applications and future directions in foot and ankle surgery.

## 2. Literature Search Method

A narrative literature review was conducted to summarize the current evidence regarding WBCT-based 3D analysis and semiautomated segmentation techniques in foot and ankle deformities. Electronic databases including PubMed, Google Scholar, and Web of Science were searched for studies published up to January 2026 using combinations of the following keywords: “weight-bearing computed tomography”, “WBCT”, “three-dimensional analysis”, “3D”, “segmentation”, “foot deformity”, “flatfoot”, “progressive collapsing foot deformity”, “cavovarus”, “Charcot-Marie-Tooth”, “hallux valgus”, and “ankle osteoarthritis”. Additional relevant studies were identified by screening the reference lists of retrieved articles.

Original clinical studies, observational studies, validation studies, and review articles published in English were considered eligible. Conference abstracts, case reports, technical notes without clinical applications, and non-English publications were excluded. Particular emphasis was placed on studies investigating clinical applications of WBCT-based 3D measurements and semiautomated segmentation techniques.

## 3. WBCT and Semiautomated Segmentation Technique for 3D Foot Analysis

**Figure 1:**
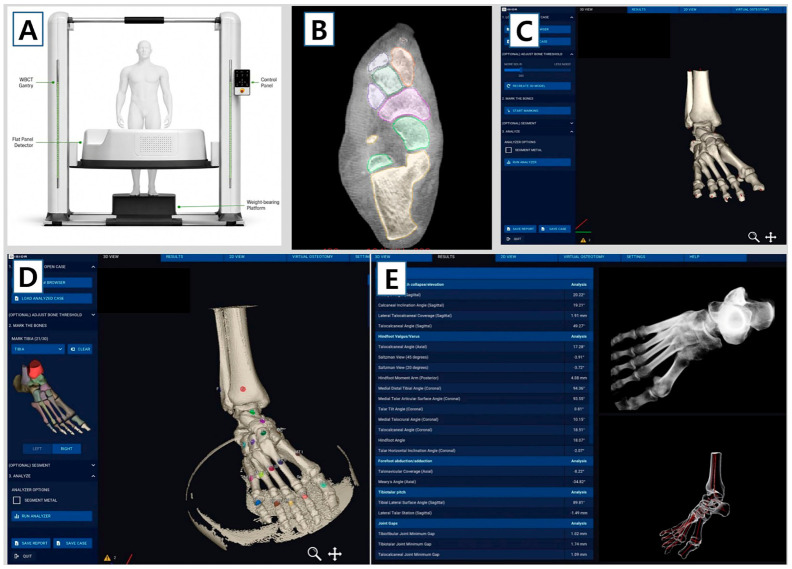
Overview of the study workflow of WBCT and semiautomated segmentation techniques for 3D foot analysis. (**A**) WBCT image scan of the patient. (**B**) Export of axial DICOM datasets from the raw imaging data (slice thickness < 0.7 mm). (**C**) 3D reconstruction of the CT images. (**D**) Manual segmentation and labeling of individual bones using 3D analysis software. (**E**) Automated analysis of inter-axis angular parameters in the sagittal, axial, and coronal planes for comprehensive 3D alignment assessment.

All patients undergo 3D WBCT for image acquisition. To achieve consistent and reproducible imaging results, patient positioning is standardized across all examinations. As shown in Figure 1A, patients stand inside the CT machine in a natural upright position under either unipedal or bipedal weightbearing conditions, depending on the CT scanner protocol.

Any comprehensive quantitative analysis of skeletal architecture using CT imaging requires precise reconstruction of bone models from sequential image slices. In practice, this process involves identifying and following the contour of each relevant bone throughout all slices, namely segmentation, to enable slice-by-slice generation of the final 3D bone model [14]. Numerous software platforms have been developed for this purpose. Ideally, such software should facilitate accurate and efficient generation of patient-specific 3D bone reconstructions. Among currently available tools for 3D skeletal reconstruction, Bonelogic software (Paragon28, Helsinki, Finland) is one of the most widely used platforms that provides semiautomated segmentation of foot bones [15]. The software rapidly and automatically determines multiple anatomical axes within the reconstructed 3D bone models, enabling accurate and reliable calculation of alignment parameters [15].

Semiautomated 3D radiographic measurements are obtained from raw Digital Imaging and Communications in Medicine (DICOM) data acquired during WBCT scanning [3,5,16]. Raw DICOM data acquired from axial CT image slices with a thickness of less than 0.7 mm are required for software-based 3D analysis. The software automatically generates a 3D isosurface representation of the osseous structures, requiring the operator only to place at least one marker on each visible bone for labeling purposes. After manual identification and registration of each osseous structure, the software automatically calculates inter-axis angular measurements for 3D evaluation of sagittal, coronal, and axial alignment. This platform has been previously validated and is recognized as a reliable tool in foot and ankle research [8,11].

According to the established literature, the evaluated foot and ankle parameters are classified into the following categories: hindfoot–forefoot relationships, hindfoot alignment parameters, coronal plane relationships, intra-forefoot relationships, and tarsometatarsal relationships [3,16]. Sagittal alignment of the hindfoot is evaluated using the sagittal Meary angle and calcaneal inclination angle. Hindfoot alignment in the axial plane is assessed using the talonavicular angle and axial Meary angle, whereas coronal plane alignment is evaluated using the talar tilt and Saltzman view angle.

Other commercially available software platforms have recently become available for 3D skeletal segmentation and morphologic analysis of the foot and ankle [9]. CubeVue (CurveBeam AI, Warrington, PA, USA) is specifically integrated with WBCT systems and provides rapid visualization of multiplanar foot alignment together with advanced weightbearing assessment, making it particularly suitable for routine clinical practice [17]. In contrast, Materialise Mimics (Materialise NV, Leuven, Belgium) is a general-purpose medical image processing platform that enables highly detailed manual and semiautomated segmentation, surgical simulation, finite element modeling, and patient-specific implant design [18,19]. Each platform offers distinct advantages depending on the intended clinical or research application. Dedicated foot and ankle software packages, such as Bonelogic and CubeVue, emphasize workflow efficiency and automated quantitative measurements for routine clinical assessment [20], whereas more versatile platforms such as Mimics provide greater flexibility for complex image processing and research applications [19]. However, differences in segmentation algorithms, automation level, and measurement definitions may result in minor variability among software platforms [11]. Since no comparative studies have established the superiority of one segmentation platform over another, further comparative studies comparing these software platforms are warranted.

## 4. Normal Foot 

In a previous study, Zaidi et al. [3] reported a comprehensive normative value of foot and ankle using WBCT-based semiautomated 3D measurements in 100 asymptomatic European subjects. Their work established essential reference values of foot across sagittal, axial, and coronal planes, thereby providing a standardized framework for defining normal 3D foot morphology in Western populations. These normative values of the foot are important because they can serve as reference standards for comparison with foot deformities such as flatfoot and cavovarus foot, thereby enabling more in-depth and meaningful research.

Similarly, to date, the majority of reference data regarding foot and ankle morphology have been established from Western populations, which may fail to account for important ethnic differences present in other populations [21]. In particular, previous studies have demonstrated that East Asian populations possess distinct foot and ankle morphometric characteristics compared with Western population [22,23]. 

**Figure 2:**
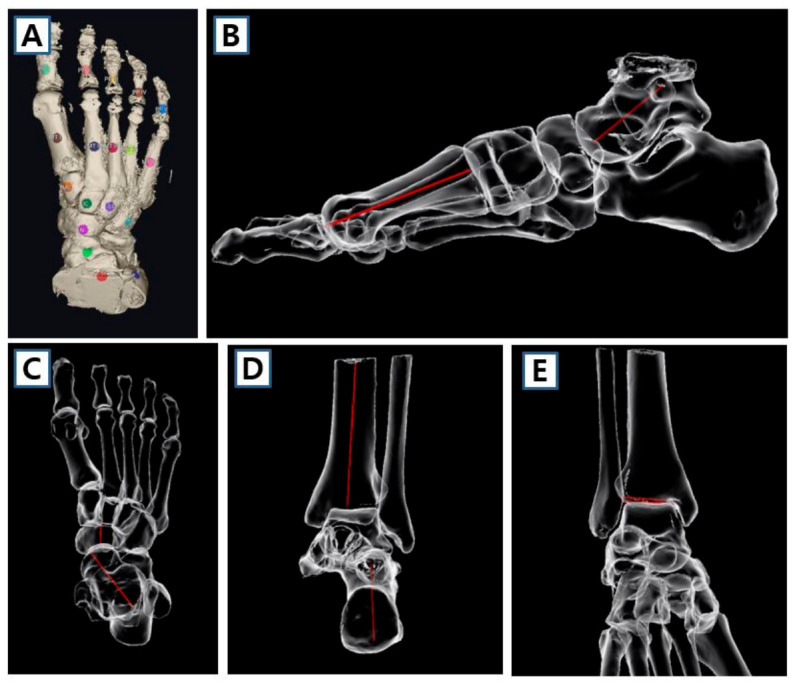
WBCT-based automated segmented images from a normal Korean cohort. (**A**) Manual labeling of each individual bone. Automated generation of segmental bone axes (red lines) for 3D assessment, including the (**B**) sagittal Meary angle, (**C**) talonavicular angle, (**D**) Saltzman view angle, and (**E**) talar tilt angle.

A recent study by Moon et al. [16] established population-specific reference values using 3D WBCT in an asymptomatic Korean cohort and compared these findings with previously established European data [3]. The Korean reference cohort consisted of 39 asymptomatic adults without previous foot surgery or clinically significant foot deformity. Individuals with hallux valgus, flatfoot, cavovarus deformity, ankle osteoarthritis, or poor image quality were excluded. WBCT images were obtained under standardized bilateral weightbearing conditions, and semiautomated segmentation was performed using Bonelogic version 2.1 software. Detailed demographic characteristics and imaging protocols have been reported previously by Moon et al. [16]. Among the evaluated measurements, 13 parameters differed significantly between the Korean and European groups (Table 1). Relative to the European cohort, Korean data exhibited significantly lower sagittal Meary angles, higher talonavicular angles, and lower Saltzman view angles. These radiographic characteristics correspond to a lower medial longitudinal arch, greater forefoot abduction, and increased hindfoot valgus, respectively (Figure 2). Overall, the current findings indicate that asymptomatic Korean feet demonstrate a stronger tendency toward flatfoot morphology, although not necessarily toward progressive collapsing foot deformity, relative to European populations. These results are in agreement with earlier anthropometric investigations reporting that East Asian populations tend to exhibit lower medial arch profiles and wider foot morphology compared with Caucasian populations [24]. The observed differences in 3D alignment between the two ethnic groups may be attributable to a complex interaction of genetic predisposition and environmental factors, particularly lifestyle-related influences [25]. For example, whereas indoor footwear use is common in Western societies, traditional Korean and other Asian lifestyles frequently involve barefoot activities within the home as well as floor-based sitting postures [26].

Detailed methodology is available in the study by Moon et al. [16].

## 5. Flatfoot Deformity

Flatfoot deformity, commonly referred to as pes planus, is a prevalent structural abnormality that affects the biomechanical alignment of the foot and ankle [27]. Flatfoot is characterized by a complex 3D deformity that encompasses hindfoot valgus malalignment, collapse of the medial longitudinal arch, and abduction of the midfoot [28,29]. Despite considerable variability in its underlying causes and clinical severity, flatfoot has frequently been linked to impaired gait mechanics and compromised dynamic function, potentially resulting in pain, functional limitations, and diminished quality of life. More recently, the term progressive collapsing foot deformity (PCFD) has been adopted to better characterize the progressive and multiplanar nature of this condition [4]. 

However, characterization of hindfoot and midfoot malalignment of PCFD using conventional weightbearing radiographs is often challenging. Shakoor et al. [30] demonstrated a strong correlation between measurements obtained from weightbearing radiographs and those derived from WBCT. Nevertheless, several parameters showed significant discrepancies between the two modalities, with conventional radiographic evaluation tending to underestimate deformity severity compared with WBCT assessment. Similar findings had previously been reported in studies involving pathologic feet [31]. 

**Figure 3:**
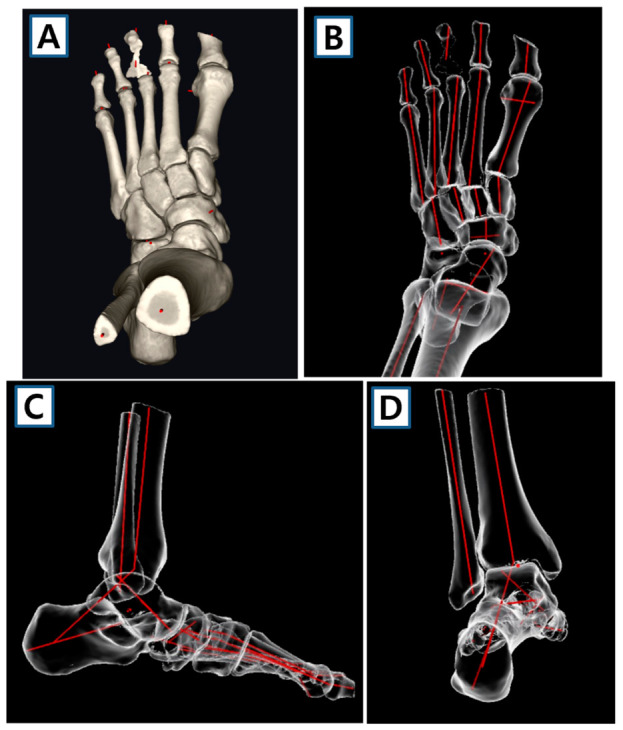
WBCT-based automated segmentation and 3D analysis of flatfoot deformity. (**A**) Reconstructed 3D image of the foot. Automated generation of segmental bone axes (red lines) for 3D assessment in the (**B**) axial, (**C**) sagittal, and (**D**) coronal planes.

WBCT enables semiautomated 3D quantification of PCFD, allowing a more precise evaluation of the deformity than that achieved with standard plain radiographic imaging or 2D CT measurements [32]. Three-dimensional angular measurements derived from reconstructed bone models demonstrate the marked global osseous malalignment associated with PCFD (Figure 3) [15]. Recently, Krähenbühl et al. [32] employed semiautomated 3D measurements to compare healthy feet with those affected by PCFD, evaluate the correlations among individual deformity parameters in PCFD, and identify measurements that may assist in distinguishing flexible from rigid feet. In this study, WBCT scans obtained from 20 patients with PCFD treated—comprising 10 cases of flexible deformity and 10 cases of rigid deformity—were compared with scans from 30 healthy controls. Semiautomated 3D analysis was used to quantify several anatomical parameters, including talar tilt, hindfoot moment arm, talocalcaneal angle (TCA; axial and lateral), talonavicular coverage (TNC), and talocalcaneal overlap (TCO). In addition, the presence of medial facet subluxation and sinus tarsi or subfibular impingement was evaluated. The results demonstrated significant differences between healthy individuals and patients with PCFD for all measured parameters except the TCA in both axial and lateral planes. Among patients with PCFD, the axial TCA showed a significant correlation with TNC. Furthermore, the combination of increased TCO and sinus tarsi impingement was associated with a higher likelihood of classifying the deformity as rigid. The study suggested that realignment arthrodesis may be considered in cases of increased TCO combined with sinus tarsi impingement, as osteotomies alone may not provide sufficient correction of the deformity. Despite these promising findings, the study included only 20 patients with PCFD and was conducted at a single institution. Therefore, the proposed radiographic thresholds should be interpreted cautiously until validated in larger multicenter cohorts.

WBCT enables comprehensive assessment of foot alignment in PCFD. However, evaluation of soft tissue structures, such as the spring ligament complex and tibialis posterior tendon, remains limited with WBCT alone [33]. The recent study by Andres et al. [33] investigated the association between osseous findings on WBCT and soft tissue abnormalities identified on magnetic resonance imaging (MRI). In this observational study, a consecutive series of 24 PCFD patients underwent both WBCT and MRI. A total of four clinically established 3D measurements were evaluated: TCO, TNC, and the sagittal and axial Meary angles. The study exhibited that a TCO of ≥15 mm or a TNC angle of ≥50° was associated with a greater than 90% probability of predicting osseous sinus tarsi impingement in the cohort. The principal findings of this study were that (1) greater foot deformity, as reflected by increased TCO, along with older age and higher body mass index, was associated with tibialis posterior tendon tears; (2) osseous sinus tarsi impingement identified on WBCT was not associated with ligamentous or tendinous tears detected on MRI; and (3) MRI overestimated the presence of osseous sinus tarsi impingement, as confirmed on WBCT, in approximately 42% of patients in their cohort. Overall, correlations between WBCT-derived 3D measurements and ligamentous or tendinous injuries detected on MRI were generally weak. The study concluded that WBCT and MRI should be regarded as complementary rather than interchangeable imaging modalities. WBCT cannot replace MRI, nor can MRI substitute for WBCT, as each provides distinct diagnostic information. Both modalities contribute unique and clinically relevant insights that may influence treatment planning and decision making in patients with PCFD.

## 6. Cavovarus Foot Deformity 

Cavovarus foot deformity is a hallmark manifestation of Charcot–Marie–Tooth (CMT) disease and frequently results in substantial gait impairment. CMT is the most common inherited peripheral neuropathy, affecting both motor and sensory nerves and resulting in a broad spectrum of clinical manifestations, including muscle wasting and sensory deficits in the upper and lower extremities [34]. An estimated 2.8 million individuals worldwide are affected by CMT, with a reported prevalence of approximately 1 in 2500 individuals in the United States [34]. Although symptoms typically develop during childhood, disease onset may occur later in life in some patients [35]. More than 70% of individuals with CMT develop a cavovarus foot deformity, making CMT the condition most frequently associated with pes cavus [36]. 

The peroneal nerve is the most commonly affected nerve in patients with CMT. Dysfunction of the peroneal nerve leads to weakness of the tibialis anterior and peroneus brevis muscles, whereas their antagonists, the peroneus longus and tibialis posterior, remain relatively preserved. Consequently, cavovarus deformity develops as a result of progressive muscular imbalance and is characterized by a combination of hindfoot varus, plantarflexion of the first metatarsal, and inversion and supination of the midfoot [34].

Radiographic evaluation of cavovarus deformity is challenging because of its complex 3D nature. In particular, midfoot inversion and supination frequently result in metatarsal stacking, making accurate assessment difficult using conventional standing plain radiographs or 2D CT [5,8]. These limitations often contribute to reduced inter-observer reliability. Recent advances in WBCT and 3D automated analysis software have enabled more comprehensive and reproducible characterization of CMT foot deformities [5,8]. WBCT is particularly useful for evaluating hindfoot alignment and ankle joint congruity in cavovarus foot, while automated analysis software allows objective measurement of angular relationships between the central axes of individual bones, thereby improving both measurement accuracy and reliability [8]. 

Recently, Sangoi et al. [8] compared manual measurements obtained from 2D WBCT images with automated measurements derived from 3D models in a cohort of 16 normal feet and 16 cavovarus feet from patients with CMT disease. Six alignment parameters were analyzed, including the Meary angle in the axial plane, the forefoot arch angle in the coronal plane, and the Meary angle, calcaneal pitch, and cuneiform- and navicular-to-floor distances in the sagittal plane. Manual measurements were performed on conventional 2D WBCT images, whereas 3D measurements were generated using Bonelogic software. The investigators found no significant measurement bias between the two techniques in normal feet, indicating that automated 3D assessment provides results comparable to traditional manual methods when foot alignment is preserved. In contrast, among cavovarus feet, the automated analysis identified greater sagittal-plane deformity and less axial-plane deformity than the corresponding manual measurements. Overall, the findings suggest that automated 3D techniques can reliably evaluate bony alignment in normal feet while providing a different characterization of deformity in cavovarus feet. The authors proposed that these discrepancies are likely attributable to the complex rotational abnormalities commonly present in cavovarus deformities, which may not be fully captured using manual measurements derived from 2D image slices. Nevertheless, the relatively small sample size and inclusion of only CMT-related cavovarus deformities may limit the generalizability of these findings to other causes of cavovarus deformity.

The configuration of the midfoot in CMT foot, which connects hindfoot varus to forefoot valgus, remained incompletely understood. The precise origin of the deformity—whether it arises primarily from intrinsic bony abnormalities or from altered intersegmental articulations—was not fully established. Michalski et al. [37] conducted a 3D analysis of hindfoot osteology in patients with CMT and identified distinct morphological differences, including a C-shaped calcaneus, compared with healthy controls. However, additional evaluation of segmental osseous and articular malalignment was necessary to clarify the mechanisms underlying the overall deformity pattern. To address this issue, Tonya et al. [10] utilized WBCT combined with segmentation techniques to compare 21 patients with CMT and 20 healthy volunteers. By decomposing the complex multiplanar deformity into its individual anatomical segments, the investigators were able to quantify both the location and magnitude of abnormal alignment with high precision. Their analysis demonstrated significantly greater axial-plane adduction and coronal-plane rotational deformity at the talonavicular joint (TNJ) in patients with CMT than in controls. They suggested that the TNJ represents a key driver of midfoot malalignment and support the use of targeted soft-tissue release at this level to achieve midfoot neutralization and derotation as an initial step in deformity correction.

A precise understanding of the anatomical location and distribution of deformities in cavovarus feet is essential, as it can facilitate more accurate surgical planning and help direct corrective procedures toward the primary sites of malalignment. The investigation conducted by Ranjit et al. [38] evaluated coronal-plane deformities in cavovarus feet associated with CMT disease using WBCT and semiautomated 3D segmentation software. Thirty CMT cavovarus feet were compared with 30 matched control feet. The authors found that the most pronounced coronal-plane deformity occurred at TNJ, where the navicular was positioned in approximately 23° greater supination relative to the talus than in normal feet, reflecting a characteristic “twisting” pattern of the foot. This deformity was partially offset by distal pronation, with the naviculocuneiform joints serving as the primary site of compensatory pronation. Consequently, the study demonstrated that, in addition to hindfoot varus, substantial supination occurs at the TNJ, whereas compensatory pronation develops at the naviculocuneiform joint. Despite this partial compensation, the overall foot remains supinated relative to the ground. 

**Figure 4:**
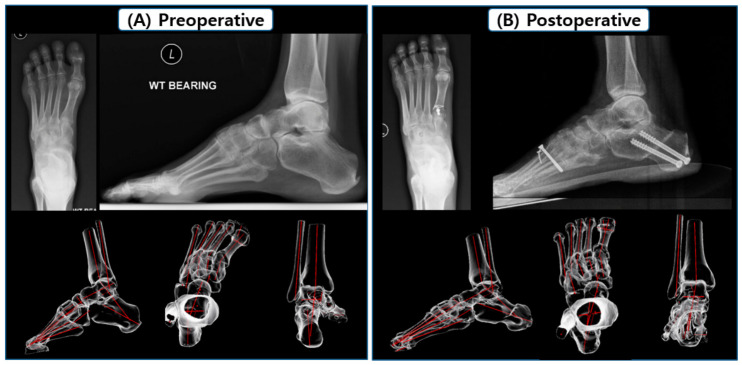
Preoperative and postoperative imaging findings of the left foot in Charcot–Marie–Tooth patient who underwent joint-sparing surgery. (**A**) Preoperative and (**B**) postoperative standing foot radiographs, WBCT-based reconstructed 3D models demonstrating the segmental bone axes (red lines) automatically generated by the software for multiplane alignment analysis.

Building on these previous findings, Song et al. [5] demonstrated the utility of WBCT combined with 3D semiautomated analysis software for the multidimensional assessment of preoperative and postoperative foot alignment in CMT patients (Figure 4). In their study, 29 patients who underwent joint-preserving reconstruction were evaluated using bone segmentation analysis of preoperative and postoperative WBCT images. The results showed that joint-preserving procedures effectively corrected deformities across the sagittal, coronal, and axial planes. In comparison with previously reported normative measurements derived from a European cohort [3], joint-preserving surgical interventions could correct foot alignment to levels closely matching those reference standards. Taken together, these studies highlight a growing trend in contemporary musculoskeletal research: the true clinical utility of WBCT-based 3D assessment lies not merely in identifying pathologic abnormalities, but in evaluating them against population-specific normative databases [3]. Furthermore, restoration of the TNJ through soft-tissue release was identified as a key component of successful deformity correction. 

Nevertheless, although advanced imaging modalities and automated analytical tools provide valuable information for surgical planning and deformity assessment, they should not be regarded as absolute guides for osseous correction. The final decision regarding the type and extent of bony procedures should be made only after all necessary soft-tissue releases have been completed and the residual deformity has been thoroughly reassessed intraoperatively [5].

## 7. Hallux Valgus Deformity

Hallux valgus is among the most prevalent disorders involving the first ray, affecting approximately 2–4% of the general population in the United States [39]. Rather than representing a simple lateral deviation of the hallux, it is a complex 3D deformity characterized by a combination of osseous and soft-tissue abnormalities. Its development is thought to result from a progressive imbalance between the medial and lateral stabilizing structures of the first metatarsophalangeal joint. Typical pathoanatomical features include varus deviation of the first metatarsal, valgus angulation and pronation of the hallux, prominence of the medial eminence, and transfer lesions such as plantar callosities beneath the lesser metatarsal heads [39,40].

Surgical intervention is often indicated when nonoperative treatment fails to adequately alleviate symptoms. The large number of operative procedures described for hallux valgus correction—exceeding 100 techniques—reflects both the complexity of the deformity and the ongoing efforts to optimize surgical outcomes [41]. In recent years, minimally invasive surgery (MIS) has emerged as an increasingly popular approach for deformity correction [42,43,44,45]. Since successful correction depends on restoring anatomy as close to normal as possible, an accurate 3D assessment of the deformity is essential for surgical planning.

**Figure 5:**
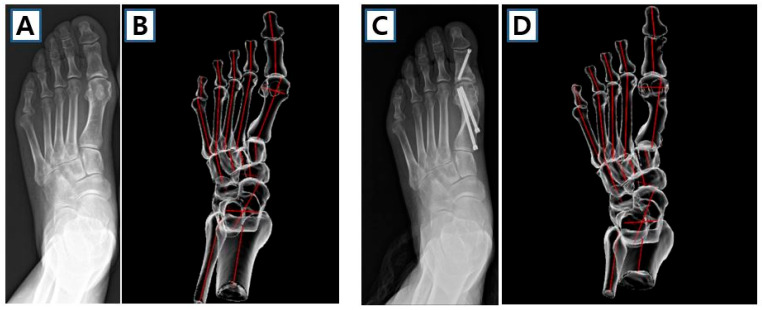
WBCT-based automated 3D analysis of hallux valgus deformity (**A**,**B**) before and (**C**,**D**) after surgery. (**A**) Preoperative weightbearing anteroposterior radiograph demonstrating a moderate hallux valgus deformity. (**B**) Preoperative automated 3D analysis showing the bone axes used for alignment assessment. (**C**) Weightbearing anteroposterior radiograph obtained 12 months after minimally invasive hallux valgus correction. (**D**) Postoperative automated 3D analysis demonstrating the reconstructed bone axes (red lines) and corrected alignment.

Recent studies have demonstrated that WBCT provides a more accurate representation of hallux valgus deformity than conventional CT imaging [46]. Using WBCT combined with dedicated 3D segmentation software, sagittal, axial, and coronal alignment parameters of hallux valgus foot can be automatically quantified (Figure 5). The study by Wang et al. [47] used WBCT to evaluate first-ray deformities as well as midfoot and hindfoot alignment in patients with hallux valgus. Conventional 2D radiographic parameters were assessed semi-automatically, while additional WBCT measurements were obtained manually. Hindfoot and midfoot parameters included foot and ankle offset, talar posterior and middle facet morphology, and the forefoot arch angle. First-ray assessments comprised first metatarsal rotation, sesamoid rotation angle, hallucal pronation angle, and sesamoid position, all of which were measured using previously validated techniques. The analysis demonstrated significant associations among metatarsosesamoid complex malalignment, coronal-plane pronation of the distal first ray, and conventional axial-plane hallux valgus deformities. In addition, both axial and sagittal Meary angles showed significant correlations with the hallux valgus angle, whereas no significant relationship was observed with the intermetatarsal angle.

Jasper et al. [48] evaluated the outcomes of the LapiCotton procedure using WBCT and 3D segmentation techniques. The LapiCotton procedure, recently introduced by de Cesar Netto et al. [49], combines the biomechanical advantages of a Cotton osteotomy with those of a modified Lapidus arthrodesis. The technique addresses medial column collapse by fusing the first tarsometatarsal joint with a dorsal wedge distraction allograft. By preserving first-ray length and plantarflexing the distal first ray, the procedure restores the medial longitudinal arch while maintaining the tripod alignment of the foot. In addition, it enables correction of transverse-plane and rotational deformities commonly associated with hallux valgus. In their study, Jasper et al. [48] investigated the effectiveness of the LapiCotton procedure in patients with hallux valgus accompanied by medial longitudinal arch collapse. Postoperative assessment using semiautomated WBCT measurements demonstrated significant improvements in hallux valgus angle, intermetatarsal angle, and sagittal Meary angle after the LapiCotton procedure. These findings indicated that the LapiCotton procedure provided reliable correction of both hallux valgus deformity and concomitant medial longitudinal arch collapse.

## 8. Anke Osteoarthritis

Ankle osteoarthritis (OA) is a prevalent degenerative condition, and chronic ankle instability is recognized as one of its major etiologic factors [8]. Persistent lateral ligament insufficiency can lead to varus ankle instability and anterior translation of the talus, both of which are considered key contributors to the development and progression of ankle OA [50]. Varus ankle OA represents one of the most common forms of ankle OA and it develops as a consequence of uneven load distribution across the tibiotalar joint caused by varus malalignment and is typically characterized by progressive cartilage degeneration involving the medial talar dome and narrowing of the medial clear space within the ankle mortise [51,52].

Previously, Kim et al. [51] utilized WBCT to evaluate axial-plane talar alignment in patients with varus ankle OA. Their findings demonstrated that internal rotation malalignment of the talus was a common feature in this patient population. Moreover, the prevalence of talar internal rotation increased with disease severity. Building upon these findings, Song et al. [50] demonstrated that the abnormal internal rotation of the talus identified on axial CT in patients with mild to moderate varus ankle OA was significantly improved following supramalleolar osteotomy. These findings suggest that ankle OA is not merely a coronal-plane deformity characterized by varus or valgus malalignment, but rather a complex 3D pathology that also involves axial rotational abnormalities. Hence, comprehensive 3D imaging assessment is essential for accurately evaluating the deformity and guiding appropriate treatment strategies.

The use of automatic 3D measurements represents an emerging approach that may facilitate a more precise evaluation of hindfoot and midfoot alignment through WBCT [53]. Recently, Kvarda et al. [54] investigated the reliability and clinical utility of automated 3D measurements in patients with posttraumatic end-stage ankle OA. The study included 72 patients with end-stage ankle OA and 20 healthy controls. Seven alignment parameters—the medial tibial articular surface angle, medial tibiotalar angle, talar tilt, hindfoot alignment angle, tibial lateral surface angle, and the lateral and axial TCA—were assessed using both conventional weightbearing radiographs and WBCT-based 3D measurements to compare the reliability and clinical utility of the two methods. As a result, the study demonstrated that automated 3D measurements showed higher inter- and intra-observer reliability than conventional 2D assessments, while maintaining comparable accuracy in both OA and healthy ankles. Furthermore, the study found that inframalleolar compensatory alignment occurred more frequently in patients with varus supramalleolar or intra-articular deformities than in those with valgus deformities. Overall, the study demonstrated that the 3D assessment technique enabled accurate and observer-independent evaluation of hindfoot and midfoot deformities in ankle OA. Accurate characterization of the location and magnitude of ankle deformity using these measurements may have important implications for treatment planning in patients with end-stage ankle OA. Additional multicenter validation studies are warranted before routine clinical implementation.

## 9. Clinical Relevance of WBCT-Based Three-Dimensional Analysis

Although WBCT-based 3D analysis provides highly detailed quantitative information regarding skeletal alignment, its clinical value ultimately depends on whether these measurements improve patient management and surgical outcomes. Beyond simply replacing conventional radiographic measurements, WBCT enables a more comprehensive understanding of multiplanar deformities and provides objective parameters that may influence diagnosis, surgical planning, and postoperative evaluation.

From a surgical perspective, WBCT-derived parameters have shown particular value for preoperative planning. In patients with cavovarus deformity, semiautomated segmentation has identified the talonavicular joint as the principal site of rotational malalignment, thereby providing surgeons with a more precise anatomical target for corrective procedures [5,10,38]. Similarly, in PCFD, 3D measurements such as talocalcaneal overlap and talonavicular coverage have been shown to differentiate flexible from rigid deformities, thereby assisting surgical decision making regarding joint-preserving osteotomy versus arthrodesis [32].

WBCT also offers considerable advantages for postoperative assessment. Unlike conventional radiographs, which primarily evaluate correction in individual planes, automated 3D analysis simultaneously quantifies changes in sagittal, coronal, and axial alignment [5]. Previous studies have demonstrated substantial restoration of multiplanar alignment following joint-preserving reconstruction for cavovarus deformity [5] and after LapiCotton procedures for hallux valgus [48], suggesting that WBCT provides an objective method for evaluating the quality of surgical correction.

Nevertheless, evidence directly linking WBCT-derived parameters with patient-reported outcome measures (PROMs), including pain, functional recovery, and patient satisfaction, remains relatively limited. Most currently available studies primarily focus on radiographic correction and measurement reliability rather than long-term clinical outcomes. Future prospective investigations integrating WBCT-derived alignment parameters with validated PROMs, such as the Foot and Ankle Outcome Score (FAOS), American Orthopaedic Foot & Ankle Society (AOFAS) score, PROMIS, and Visual Analog Scale (VAS), will be essential for establishing the true clinical utility of automated 3D assessment. Such studies may further determine whether specific three-dimensional alignment parameters can serve as prognostic biomarkers for surgical success or predictors of recurrence.

## 10. Overall Evidence Synthesis

Across the currently available literature, WBCT-based semiautomated segmentation has consistently demonstrated superior characterization of multiplanar deformities compared with conventional radiography. This advantage is particularly evident in disorders involving rotational malalignment, including PCFD, cavovarus deformity, hallux valgus, and ankle osteoarthritis. Despite variations in study design and evaluated parameters, most investigations reported improved measurement reliability and enhanced visualization of complex skeletal relationships.

Nevertheless, the existing evidence remains limited by relatively small sample sizes, single-center designs, heterogeneous measurement protocols, and the lack of standardized software platforms. Furthermore, relatively few studies have investigated the association between WBCT-derived measurements and long-term patient-reported outcomes.

Overall, current evidence supports WBCT-based three-dimensional analysis as a promising adjunct for diagnosis and surgical planning rather than a replacement for conventional imaging. Future multicenter prospective studies are needed to establish standardized measurement protocols and determine the prognostic value of WBCT-derived parameters.

## 11. Limitations and Future Directions

Despite its considerable advantages, WBCT-based 3D analysis remains associated with several important limitations that should be considered before widespread clinical implementation.

First, although WBCT provides substantially improved visualization of osseous alignment compared with conventional radiography, it remains primarily a bone imaging modality [20]. Direct evaluation of ligaments, tendons, cartilage, muscles, and synovial structures is limited because of the inherently low soft tissue contrast of CT imaging. Although several osseous findings, including sinus tarsi narrowing, joint subluxation, and secondary degenerative changes, may indirectly suggest associated soft tissue pathology, MRI remains the reference standard for evaluating tendon tears, ligament injuries, cartilage degeneration, and inflammatory abnormalities [33]. Accordingly, WBCT should be regarded as complementary rather than a replacement for MRI, particularly in patients with complex foot and ankle disorders involving both osseous and soft tissue pathology.

Second, although numerous studies have demonstrated superior inter-observer and intra-observer reliability of WBCT-derived automated measurements compared with conventional radiographs [11], measurement reproducibility may still vary depending on image quality, segmentation accuracy, and operator experience. Furthermore, most currently available validation studies have been performed in specialized centers with considerable experience using dedicated segmentation software. Additional multicenter validation studies are therefore warranted to confirm reproducibility across different institutions and imaging systems.

Third, accessibility remains an important challenge. Dedicated WBCT scanners are currently available primarily in specialized foot and ankle centers, and their widespread implementation may be limited by equipment cost and institutional resources [20]. Furthermore, several commercial segmentation platforms are currently available, including Bonelogic and Materialise Mimics. These software packages differ considerably in automation level, segmentation algorithms, workflow, processing time, and regulatory approval. Consequently, measurement values and workflow efficiency may not be directly interchangeable across different platforms, highlighting the need for software-independent validation and standardization.

Finally, recent advances in artificial intelligence (AI) and deep-learning algorithms are expected to further transform automated bone segmentation. Emerging AI-based approaches have demonstrated promising accuracy for fully automated segmentation, landmark detection, and anatomical measurements while substantially reducing processing time and operator dependency. Future integration of AI with WBCT may facilitate real-time automated deformity analysis, surgical simulation, patient-specific surgical planning, and digital twin technologies. Furthermore, future research should increasingly focus on integrating WBCT-derived three-dimensional parameters with long-term clinical outcomes and machine learning-based predictive models, thereby moving beyond descriptive morphologic assessment toward precision medicine in foot and ankle surgery.

## 12. Conclusions

Recent studies have consistently demonstrated the clinical value of WBCT-based semiautomated segmentation technique across a wide spectrum of foot and ankle deformities. By providing a more comprehensive and accurate understanding of complex osseous alignment and joint relationships than conventional imaging modalities, these technologies have enhanced both deformity assessment and surgical decision making. As WBCT-based automated 3D segmentation analysis continues to evolve and become more widely accessible, it is anticipated that their integration into clinical practice will play an increasingly important role in the diagnosis, treatment planning, and outcome evaluation of foot and ankle disorders. Consequently, familiarity with these advanced imaging tools and their appropriate application will become an essential component of contemporary foot and ankle practice for orthopedic surgeons.

## Figures and Tables

**Table 1:** Comparison of foot and ankle parameters between Korean and European cohorts [16].

	Korean Cohort (*n* = 39) ^a^	European Cohort (*n* = 100) ^a^	Mean Difference ^a^	*p*-Value
Hindfoot–forefoot relationship				
Meary’s Angle (Sagittal)	−12.20 ± 7.67	−4.26 ± 6.06	−7.94	<0.001 **
Calcaneal Inclination Angle (Sagittal)	17.91 ± 5.06	21.86 ± 5.22	−3.95	<0.001 **
Talonavicular Angle (Axial)	40.67 ± 7.07	30.75 ± 8.23	9.92	<0.001 **
Meary’s Angle (Axial)	23.79 ± 9.01	11.86 ± 9.56	11.93	<0.001 **
Hindfoot alignment angles				
Saltzman View (45 degrees)	−4.04 ± 7.46	4.45 ± 5.78	−8.49	<0.001 **
Saltzman View (20 degrees)	−0.13 ± 9.11	9.08 ± 6.43	−9.21	<0.001 **
Coronal plane relation				
Medial Distal Tibial Angle (Coronal)	90.82 ± 3.66	92.22 ± 2.86	−1.40	0.018 *
Talar Tilt Angle (Coronal)	−0.47 ± 1.29	0.3 ± 1.02	−0.77	<0.001 **
Intra-forefoot relationship				
1st–2nd Intermetatarsal Angle (Axial)	12.55 ± 2.10	11.53 ± 1.73	1.02	0.004 *
1st–2nd Intermetatarsal Angle (Sagittal)	2.03 ± 3.04	3.23 ± 2.04	−1.20	0.007 *
1st–5th Intermetatarsal Angle (Axial)	30.77 ± 3.86	27.8 ± 3.53	2.97	<0.001 **
1st–5th Intermetatarsal Angle (Sagittal)	−13.30 ± 3.60	−11.72 ± 3.65	−1.58	0.023 *
Tarsometatarsal relationship				
3rd Tarsometatarsal Angle (Axial)	−17.52 ± 3.14	−18.95 ± 1.97	1.43	0.002 *

^a^ Values are presented as mean ± standard deviation; * *p* < 0.05. ** *p* < 0.001.

## Data Availability

The data presented in this study are available in the article.
